# Inflammaging Markers in the Extremely Cold Climate: A Case Study of Yakutian Population

**DOI:** 10.3390/ijms252413741

**Published:** 2024-12-23

**Authors:** Alena Kalyakulina, Igor Yusipov, Elena Kondakova, Tatiana Sivtseva, Raisa Zakharova, Sergey Semenov, Tatiana Klimova, Elena Ammosova, Arseniy Trukhanov, Claudio Franceschi, Mikhail Ivanchenko

**Affiliations:** 1 Artificial Intelligence Research Center, Institute of Information Technologies, Mathematics and Mechanics, Lobachevsky State University, 603022 Nizhny Novgorod, Russia; yusipov.igor@gmail.com (I.Y.); elen_kondakova@list.ru (E.K.); ivanchenko.mv@gmail.com (M.I.); 2 Institute of Biogerontology, Lobachevsky State University, 603022 Nizhny Novgorod, Russia; claudio.franceschi@unibo.it; 3 Research Center of the Medical Institute, M.K. Ammosov North-Eastern Federal University, 677013 Yakutsk, Russia; tm.sivtseva@s-vfu.ru (T.S.); prn.inst@mail.ru (R.Z.); insemenov@yandex.ru (S.S.); biomedykt@mail.ru (T.K.); ammosovael@mail.ru (E.A.); 4 Mriya Life Institute, National Academy of Active Longevity, 124489 Moscow, Russia; arseniy.trukhanov@gmail.com

**Keywords:** Yakutia, inflammatory profile, cold environment, climate, deep neural network, explainable artificial intelligence

## Abstract

Yakutia is one of the coldest permanently inhabited regions in the world, characterized by a subarctic climate with average January temperatures near −40 °C and the minimum below −60 °C. Recently, we demonstrated accelerated epigenetic aging of the Yakutian population in comparison to their Central Russian counterparts, residing in a considerably milder climate. In this paper, we analyzed these cohorts from the inflammaging perspective and addressed two hypotheses: a mismatch in the immunological profiles and accelerated inflammatory aging in Yakuts. We found that the levels of 17 cytokines displayed statistically significant differences in the mean values between the groups (with minimal *p*-value = 2.06 × 10^−19^), and 6 of them are among 10 SImAge markers. We demonstrated that five out of these six markers (PDGFB, CD40LG, VEGFA, PDGFA, and CXCL10) had higher mean levels in the Yakutian cohort, and therefore, due to their positive chronological age correlation, might indicate a trend toward accelerated inflammatory aging. At the same time, a statistically significant biological age acceleration difference between the two cohorts according to the inflammatory SImAge clock was not detected because they had similar levels of CXCL9, CCL22, and IL6, the top contributing biomarkers to SImAge. We introduced an explainable deep neural network to separate individual inflammatory profiles between the two groups, resulting in over 95% accuracy. The obtained results allow for hypothesizing the specificity of cytokine and chemokine profiles among people living in extremely cold climates, possibly reflecting the effects of long-term human (dis)adaptation to cold conditions related to inflammaging and the risk of developing a number of pathologies.

## 1. Introduction

The Republic of Sakha (Yakutia) is one of the coldest permanently inhabited regions of the world with a subarctic climate characterized by a huge amplitude of air temperature fluctuations reaching 100 °C (up to −60 °C in winter, up to +40 °C in summer) and the average winter temperature in Yakutsk reaching −42 °C [1]. A large part of the population are Yakuts, an indigenous people living in the subarctic and arctic territories of Eastern Siberia [2]. The territory of the modern Sakha Republic was settled about 30,000 years ago [3], and the northeastern part of Yakutia is located on one of the main migration routes from the southern regions of the Yenisei, Amur, and Baikal to the Arctic coast and to the Americas [4]; the last wave of migration to the territory of modern Yakutia was about 2000 years ago [5]. 

Environmental variables are of tremendous importance for the development of age-related diseases, which has been accumulated in the concept of “exposome” [6,7]. Particular attention is paid to the interplay between genetics and eco-evolutionary determinants of human longevity [8]. Among others, the populations living in cold climates (the far north in Russia, northern Canada and Scandinavia, Greenland, the highlands of South America and Tibet) are understudied, primarily due to the remoteness of the location, harsh weather conditions, and related difficulties in data collection. Existing results on Siberian populations mainly address the genetics of adaptation to unfavorable environmental factors [9], such as low lipid levels due to increased energy metabolism [10], high blood pressure [11,12], cold adaptation genes related to energy and metabolic regulation [9], and cold adaptation-related single nucleotide polymorphisms [1]. These geographical factor can also influence epigenetic age acceleration [13]; in particular, we have demonstrated that it is significantly higher in Yakutia representatives than in their counterparts from Central Russia [2].

Some studies give grounds to expect specificity in inflammatory profiles of the Yakutian population. In [14], it was shown that the Yakuts are characterized by an increased IgA level (which may indicate inflammatory processes in the mucous membranes of the respiratory, digestive, and urinary systems) and increased levels of IgE (allergy). People newly arrived in the region manifested immune system activation associated with adaptive mechanisms i.e. increased IgM levels (refers to acute inflammatory process).

Changes in immune profile are an important biomarker of human health status, related to aging and age-related diseases, and some results suggest that there also exists geographic specificity that might be influenced by ethnic, climate and nutritional factors [15,16,17]. The concept of inflammaging addresses the chronic, sterile, low-grade inflammation that increases with age, subject to environmental factors and individual immunobiography, and contributes to the pathogenesis of age-related diseases [18,19,20,21]. Inflammaging can be quantified by estimating the biological age from the human immunological profile, based on pro- and anti-inflammatory cytokines. One of the best known is the iAge inflammatory model, which estimates age, based on cytokine, chemokine, and growth factor information. This model has shown strong associations of cytokine CXCL9 with age, as well as associations with multimorbidity, immune senescence, frailty and cardiovascular aging [22]. SImAge, a small immunological clock model, is a deep neural network model that estimates age from 10 immunological measures and is sensitive to kidney disease and mortality [23]. The other immunological models are Inflammatory Biologic Age [24], IMM-AGE [25], ipAGE [26], and CyClo [27]. Inflammatory clock models implement machine learning approaches, which put forth the issue of explainability of their results, especially when deep architectures are used [28]. To the best of our knowledge, inflammatory clocks have not previously been employed to characterize the regional differences in human populations

Here we present the results of inflammatory profiling of the two cohorts, a group of Yakuts and a group of residents from Central Russia, that have previously been subject to epigenetic data studies [2]. Both cohorts include participants from the general population, without acute chronic diseases. The generated data represent the levels of 32 pro- and anti-inflammatory cytokines, including 10 cytokines from the SImAge inflammatory biological clock [23]. This investigation was primarily focused on identifying discrepancies in the immunological profiles of the selected cohorts, as well as on analyzing inflammatory age acceleration. We provided a statistical analysis of differential levels of the involved cytokines and investigated if the inflammatory clocks manifest accelerated aging of the Yakutian group with respect to the Central Russian one. We also sought to construct immunological profiles by machine learning that separated the participants from the two study cohorts and developed an explainable deep classifier, highlighting cytokines that serve as the most important features. The relevance of the identified inflammatory markers to regional environmental factors was analyzed. 

## 2. Results

### 2.1. Characterization of the Participants

**Figure 1:**
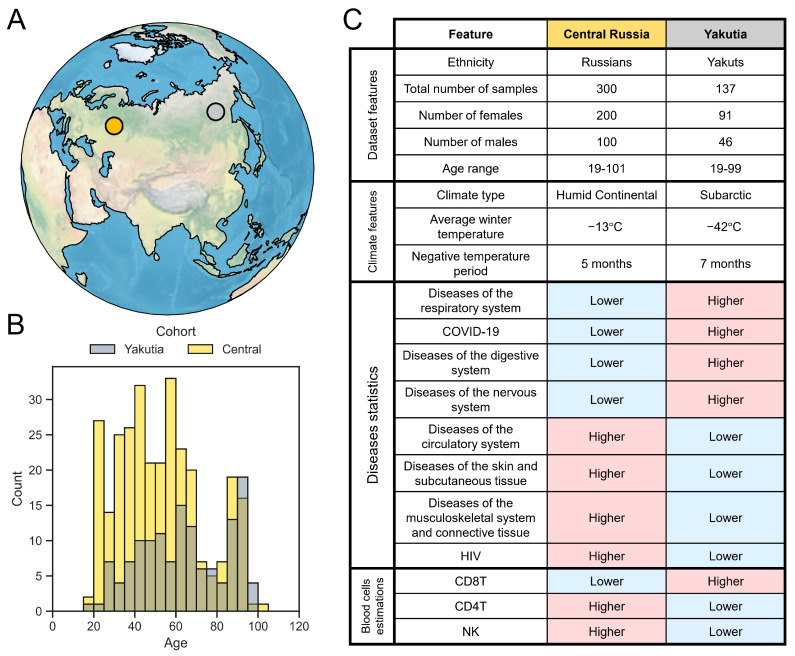
Main characteristics of the study cohorts. (**A**) Geographic schematic representation of the location of Yakutia (silver) and Central Russia (Nizhny Novgorod Oblast, gold) on a globe. (**B**) Histogram of the age distribution of participants. (**C**) Table with some features of interest in the compared cohorts—dataset and climate features, disease statistics, and blood cell estimates.

The study cohort included the groups of participants from two distant geographic regions—Yakutia (Republic of Sakha, highlighted in gray in Figure 1A) and Central Russia (Nizhny Novgorod, Vladimir, and Moscow Regions, highlighted in yellow in Figure 1A). These regions are about 5000 km apart and differ significantly in environmental conditions. The analyzed cohort included 300 samples from Central Russia (100 men and 200 women) and 137 samples from Yakutia (46 men and 91 women). In both groups the distribution by sex was uneven; the number of women was about two times greater than men. For the analysis, we used 32 common immunological biomarkers obtained using Luminex xMAP technology for blood plasma samples. The values of the analyzed biomarkers for all samples are presented in Supplementary Table S1.

The considered groups had similar and very wide age ranges—from 19 to 101 years in the Central region, from 19 to 99 years in Yakutia. The age distributions in both cohorts are presented in Figure 1B. During data collection, we paid special attention to ensuring that all age groups were represented. The Central Russia group had more young participants than Yakutia, and the number of older participants was about equal. In both regions there was a slight underrepresentation of participants in the 70–80 age group.

Yakutia and Central Russia differ in a huge number of various parameters affecting many aspects of life, despite being located within the same country (the most important in the context of this analysis are presented in Figure 1C). The main differences between the studied regions are climate and ethnicity. The Yakutia group included an indigenous population, Yakut by nationality, representing Asians, whereas the Central Russian participants had Caucasian backgrounds. Yakuts live in an extremely cold subarctic climate with an average winter temperature of about −42 °C and more than 7 months of subzero temperatures. The inhabitants of Central Russia live in a much more comfortable humid continental climate with an average winter temperature of about −13 °C and a duration of subzero temperatures of about 5 months [29]. Disease types are differentially represented in the considered regions—in particular, allergic reactions, digestive diseases and diabetes are more prevalent in Central Russia, while the incidence of viral infections and obesity is higher in Yakutia [30].

### 2.2. Identifying Differences in Inflammatory Profiles

#### 2.2.1. Differential Analysis of Cytokine Levels

**Figure 2:**
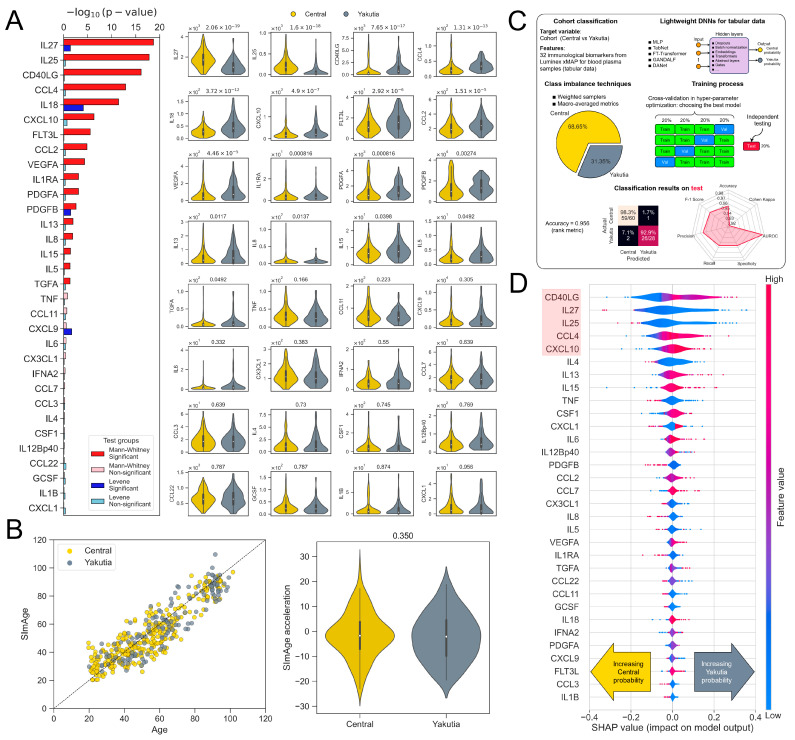
(**A**) (left) Bar plot illustrating FDR-corrected *p*-values of the Mann–Whitney U-test (red) and Levene test (blue) for each immunological biomarker when comparing the mean and variance (relative to the median) values of the distributions in the two study cohorts. (right) Violin plots showing the distributions of all immunological biomarker levels with the FDR-corrected *p*-value of the Mann–Whitney U-test. (**B**) (left) Scatter plot representing the dependence of chronological age on predicted immunological age using the SImAge model [23]. One point corresponds to one participant. MAE for Yakutia is 8.05 years, for Central Russia it is 7.14 years. Pearson correlation coefficient for Yakutia is 0.897, for Central Russia it is 0.898. (right) Violin plots representing SImAge acceleration distributions in two cohorts; Mann–Whitney U-test *p*-value is 0.35 and shows no statistically significant difference between the mean of the two distributions. (**C**) The main steps in building a deep classifier include applying lightweight deep neural networks for tabular data, techniques to overcome class imbalance, cross-validation and testing on a separate block of data. The results of the classifier on the test data demonstrate an accuracy of 0.956; a confusion matrix and spider plot with more metrics are also shown. (**D**) Results of applying explainable artificial intelligence (SHAP) to the final deep classifier. The distributions for each individual biomarker demonstrate the relationship between the biomarker level and SHAP value for all participants. The biomarker level is color coded. Movement toward negative SHAP values corresponds to an increase in the probability of predicting the Central region, while movement toward positive values corresponds to an increase in the probability of predicting Yakutia. The five most important cytokines are highlighted in red.

One approach to assessing differences in the levels of immunological parameters between representatives of the considered groups can be a statistical test (Mann–Whitney U-test) that measures, for example, the mean levels of distributions of certain cytokines and indicate the presence/absence of statistical significance. Not only mean values, but also variance, can be compared (the Levene test measures the variance of distributions relative to the median). The biomarker distributions together with the FDR-corrected *p*-values are presented in Figure 2A. Seventeen cytokines demonstrated statistically significant differences between the regions, and most of them had higher mean values in Yakutia representatives. Only four biomarkers displayed statistically significantly different distribution variances: IL27, IL18, PDGFB and CXCL9 (the first three also have different mean values), while the majority had a similar variance between the groups. Detailed statistical metrics for the distributions of all cytokines are given in Supplementary Table S2. At the same time, none of the biomarkers showed clear separability for the two populations, which means that it is not possible to determine whether a sample belongs to one group or another based on the value of a single biomarker. The commonalities in the immunomarkers profiles could be due to the factors shared between the populations, like the lifestyle, socioeconomic status and morbidities; however, the current data are insufficient to address this issue in detail. Of note, statistical tests allow a direct comparison of cytokine levels individually without taking into account the interactions between them. A deep machine learning classifier based on a number of biomarkers can help to overcome this problem (cf. Section 2.2.3).

#### 2.2.2. Immunologic/Inflammatory Clock

We also compared these two cohorts by immunological age acceleration using the SImAge model [23]. The choice of the model was data driven, since it is adapted for the employed immunological panel (cf. Materials and Methods). Although Yakuts showed accelerated epigenetic aging relative to representatives of the Central region (from 1 to 5 years depending on the epigenetic clock model) [2], they did not show significantly accelerated immunological aging (Figure 2B). This may be due to the fact that the mean levels of the three most important immunomarkers in the SImAge model (CXCL9, CCL22, and IL6) are not statistically significantly different between the considered groups. However, we note that six of the cytokines included in the SImAge model have statistically significantly different levels in Yakuts. Levels of CD40LG, CXCL10, PDGFA, PDGFB, and VEGFA are higher in Yakutia than in Central Russia, and only one, IL27, is lower in Yakutia. Since all of these parameters have a positive correlation with age [23], this may indicate some weak trend toward higher levels of inflammation in Yakuts.

#### 2.2.3. Explainable Deep Learning Classifier

Another approach to detect differences between groups is to build an explainable deep neural network classifier that both accounts for complex nonlinear dependencies in the data and highlights the most important features (levels of certain cytokines) to separate members from the considered geographic regions. Figure 2C presents the main steps of the construction of the deep classifier and its performance on the test set (details of the model construction and implemented technologies are presented in the Methods). The input features of the classifier were represented by the levels of the 32 immunological biomarkers in the blood plasma samples (the data are organized in tabular form—rows represent samples, columns represent features), the target variable was a cohort (Central or Yakutia). Since the classes (Central vs. Yakutia) were unbalanced (the number of samples in one class significantly exceeded the number of samples in another class), we leveled out these differences, reducing their impact on the classifier: the weighted sampler ensured that the Yakutia class was not underrepresented in each batch when training the model, and the macro-averaged metrics treated classes equally when calculating the results. The training process included cross-validation, during which the best hyperparameters of the machine learning models were identified, and they were tested on an independent subset of the data (samples separated from the main dataset in advance and not involved in model training). The best results were achieved by DANet neural network architecture (details given in the Methods), which discriminated between samples from Yakutia and the Central region with a high accuracy of 0.956. The other metrics of the final model and confusion matrix are presented in Figure 2C.

Complex neural network architectures are often ‘black boxes’ whose decision-making principles are hard to interpret. However, in recent years, eXplainable Artificial Intelligence (XAI) approaches have been actively developed, which allow to identify the features that have the highest impact on the final result (in our case, which biomarker values are most important for distinguishing Yakuts from Centrals) [28,31,32]. One of the most common techniques is SHAP, which utilizes game theory approaches and has a broad applicability, allowing us to explain the results of the models identifying important features for the considered classes. Figure 2D shows beeswarm plots displaying the magnitude of SHAP values for all immunological biomarkers. In our case, more negative SHAP values increased the probability of predicting the Central region and more positive values increased the probability of predicting Yakutia. In particular, higher values of CD40LG (the first in the ranking of the most important features for the classifier) increased the probability of assigning the sample to the Yakutia group, and lower values increased the probability of assigning the sample to the Central Russia group. Altogether, CD40LG, IL27, IL25, CCL4, and CXCL10 manifest the most significant impacts on the classifier results (highlighted in red in Figure 2D), while IL1B and CCL3, on the contrary, demonstrate the least contribution to group differentiation. Thus, the members of the Yakutia and Central Russia populations that we included in our data are well separated by an immunological profile using a deep classifier, although they are inseparable in terms of the levels of individual cytokines.

### 2.3. Outlook on Biological and Clinical Relevance

Next, we examined in more detail some biological functions of immunological biomarkers that are in the top of importance for separating representatives of Yakutia and Central Russia by two characteristics at the same time: SHAP values of the deep classifier and Mann–Whitney U-test of statistical significance of the difference in means of distributions.

CD40LG (sCD40L), a soluble fragment of CD40 ligand, is a type II membrane protein belonging to the tumor necrosis factor family and mainly expressed on the surface of activated CD4+ T cells [33]. CD40LG from platelets mediates thrombotic and inflammatory processes, contributing to inflammation associated with viral infections [34], which have a higher prevalence in Yakutia (COVID-19 in particular) [35] due to, among other factors, weakened nasal defenses (cold weather slows the ability to clear mucus from the nose, which makes it easier for viruses to enter the body). Higher levels of CD40LG may be associated with thrombogenesis, inflammation, atherosclerosis, coronary artery syndrome, and systemic lupus erythematosus [33,36,37]. Viliui encephalomyelitis, a neurodegenerative disease of the central nervous system, occurs only in the indigenous population of Yakutia, and elevated CD40LG levels (like in our data) can be observed not only in patients but also in healthy family members [38]. 

IL27 is a cytokine of the IL12 family that has both pro-inflammatory and anti-inflammatory effects [39]. The proinflammatory effect of IL27 is manifested in its ability to enhance the expression of inflammatory cytokines and chemokines in primary monocytes and to promote proliferation of naive CD4+ T cells (estimates of the count of monocytes and CD4T cells based on DNA methylation data are lower in Yakutia, as are the IL27 levels observed here) [2,40,41]. High IL27 levels are associated with many autoimmune diseases (rheumatoid arthritis, systemic lupus erythematosus, and multiple sclerosis) [42], which are statistically less common in Yakutia [35], and are also negatively correlated with HIV viral load [42], which is more common in Yakutia (and IL27 levels are lower there) [35]. The role of IL27 in the immune response against bacterial infections is different: at the beginning of infection, IL27 stimulates immune responses at the site of infection, while at later stages, IL27 suppresses inflammatory responses of immune cells to avoid multi-organ failure due to excessive or persistent inflammation [41]. 

IL25 (IL17E) is a cytokine of the IL17 family, originally discovered through sequence alignment of human genomic DNA. It is secreted in activated eosinophils and basophils in allergy, in epithelial cells at sites of inflammation, in keratin-forming skin cells in psoriasis, in mast cells, and in intestinal epithelial cells (tuft cells) [43]. An increase in the IL25 level contributes to the development and increased severity of skin diseases (e.g., dermatitis and psoriasis) [43], which are statistically less common in Yakutia [35]. A decrease in its expression may be observed in inflammatory bowel disease (digestive diseases are statistically twice more frequent in Yakutia), and increased levels are associated with obesity and the intensity of low-grade inflammation and may be a biomarker of cardiovascular disease risk (residents of Central Russia are statistically more likely to suffer from circulatory diseases) [35,43,44]. 

CCL4 (MIP-1b), CC motif chemokine ligand 4 or macrophage inflammatory protein-1b [45], is a highly acidic chemokine [46]. Monocytes, T and B lymphocytes, dendritic cells, neutrophils, and NK cells are among the cellular sources of CCL4 (estimates of the NK cell count according to DNA methylation data are higher in Yakutia, as well as the CCL4 levels observed here) [2,45]. Reduced CCL4 concentrations may be observed in patients with type 1 and type 2 diabetes, the incidence of both being lower in Yakutia [35,47]. However, some studies have observed increased CCL4 concentrations in patients in the prediabetic state with a tendency to decrease with insulin intake, which may indicate that CCL4 levels may differ at different stages of diabetes development or severity [47]. Elevated CCL4 levels may be associated with Parkinson’s disease, Alzheimer’s disease, and blood–brain barrier dysfunction (neurodegenerative diseases are statistically more common in Yakutia) [35,48]. 

CXCL10 (IP10), also called C-X-C motif chemokine ligand 10 or interferon γ-induced protein 10 kDa, is a major proinflammatory Th1 chemokine that is involved in the pathophysiology of multiple diseases. It is released in response to IFN-γ from CD4+, CD8+ and natural killer cells (estimates of CD8T and NK cell counts based on DNA methylation data are higher in Yakuts, as are the CXCL10 levels observed here) [2,49]. CXCL10 plays a key role in the development of respiratory symptoms in viral diseases (the population of Yakutia is more susceptible to respiratory diseases and viral infections according to statistics) [35,50]. Abnormal levels of CXCL10 were observed in body fluids of people infected not only with viruses, but also with bacteria, fungi and parasites [51]. Elevated levels of CXCL10 were observed in COVID-19 and were associated with a severe course and progression of the disease, predicting ARDS and neurological complications (Yakutia has higher levels of COVID-19) [35,52].

**Figure 3:**
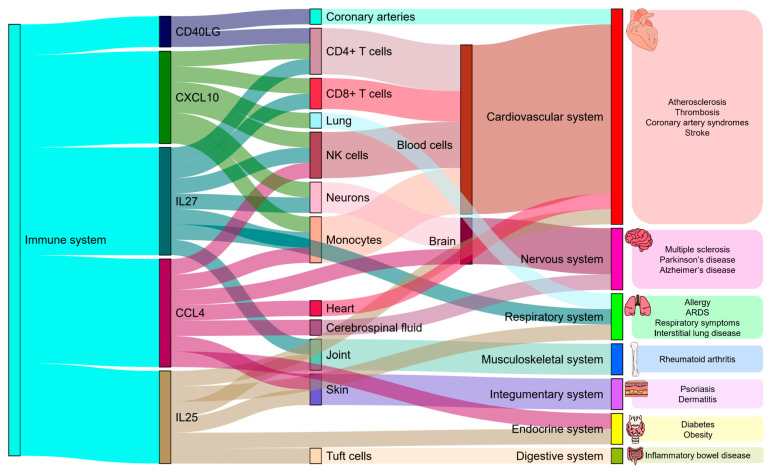
Sankey plot presenting some key relationships between immunological biomarkers and different body systems with the selected comorbidities. The immunomarkers are chosen as follows: they are important for a deep classifier and at the same time have statistically significantly different mean values of level distributions between the studied groups. The associations in the plot are based on a literature review.

The key associations of the analyzed biomarkers are summarized in Table 1 and Figure 3.

These inferences made, it should be kept in mind that this study refers to a single limited-size group of participants from Yakutia, whereas no data from other populations are available for direct comparison, to the best of our knowledge. Therefore, generalization to the entire population has to be made with caution. Since chronic diseases in the acute phase were an exclusion criterion, the possible relevance between the region-specific inflammatory profiles and diseases is discussed in the context of open source general statistics. Although we emphasize climate factors and morbidity statistics as the most pronounced differences, the other region-specific socioeconomic factors, diet, water/air quality, and so forth may also affect the immune status. The morbidity statistics for Central Russia are associated with the Nizhny Novgorod region, since the absolute majority of participants are its residents.

### 2.4. Sex-Specific Differences Inside and Between Regions

**Figure 4:**
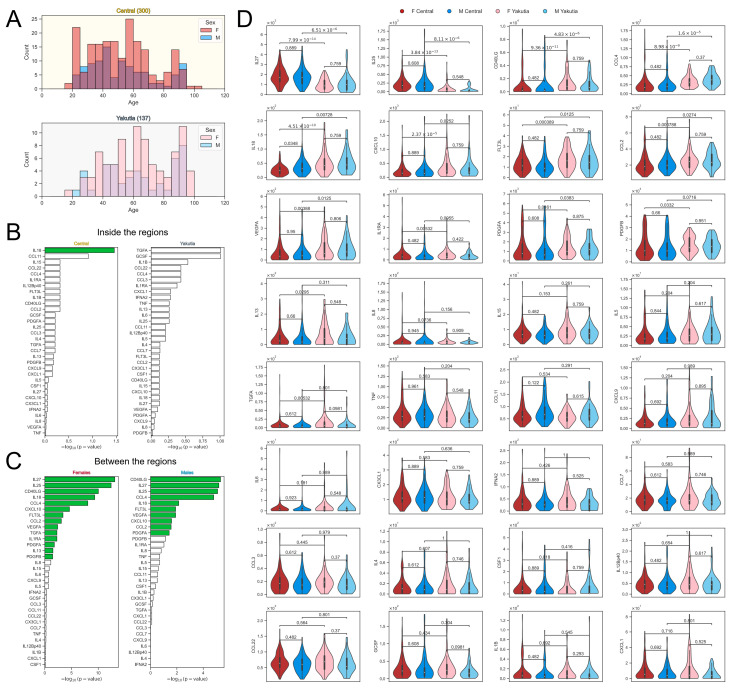
(**A**) Histograms of participant age distributions for women and men in Central Russia (top) and Yakutia (bottom). (**B**) Results of Mann–Whitney U-test with FDR-corrected *p*-values for women and men inside Central Russia (left) and Yakutia (right). Statistically significant features are shown by the green bars. (**C**) Results of the Mann–Whitney U-test with FDR-corrected *p*-values for women (left) and men (right) between the regions. Statistically significant features are shown by green bars. (**D**) Violin plots showing the distributions of all immunological biomarker levels with the FDR-corrected *p*-values of the pairwise Mann–Whitney U-tests for four groups: women and men from Central Russia and Yakutia separately.

We also additionally investigated cytokine levels separately in men and women in the studied regions. Age distributions in women and men were generally similar between regions, with both sexes represented in all age groups (Figure 4A). We first examined sex differences in immunologic profiles within each region using the Mann–Whitney U-test (Figure 4B). In Central Russia, only one cytokine, IL18, was significantly different between women and men; in Yakutia, none. Next, we compared women and men between regions (Figure 4C). Here, the differences were more significant: 14 of 32 cytokines were significantly different between the regions in women, and 10 of 32 cytokines were significantly different in men. All 10 markers that differed significantly in men also differed significantly in women. The female-specific cytokines were PDGFB, IL13, IL1RA, and TGFA. Noteworthy, among the immunomarkers differing between sexes, all 14 were also significantly different in the whole cohort study. IL5, IL15, IL8 differ when considering whole cohorts but do not differ separately in men and women. All corresponding cytokine distributions in the four studied subgroups are shown in Figure 4D.

## 3. Discussion

This paper presents the results of a study of novel data on immunological profiles (levels of 32 plasma cytokines) of a group of indigenous Yakuts that has not been previously investigated. We compared their immunological profiles with those of Central Russian counterparts who reside in a milder environment. Two approaches were employed to identify differences in immunological profiles: statistical methods and explainable deep classifiers. We found that 17 cytokines display statistically significant differences in their levels between the two groups. Most of them take higher levels in the Yakutian group. At the same time, their levels manifest strongly overlapping distributions of values, and it is impossible to discriminate between individual participants with respect to the groups based on single biomarkers. 

However, it turned out that the developed machine learning classifier based on a deep DANet neural network can distinguish the participants from Yakutia and from Central Russia, with a high accuracy (exceeding 95%). This suggests that the Yakutian and Central Russia participants are characterized by specific multi-cytokine inflammatory profiles. Explainable artificial intelligence approaches identified that the top-rated cytokines in these profiles are CD40LG, IL27, IL25, CCL4, and CXCL10. These biomarkers were also found to exhibit statistically significant differences. 

### 3.1. Clinical and Practical Implications

All of these immunomarkers are directly related to the functioning of the immune system and multiple processes associated with the activation of the immune response. The existing literature suggests that the high levels of certain cytokines are related to a higher incidence of certain diseases in the same region. Further investigation is required to check this hypothesis, involving larger cohorts from representative populations. In particular, the increased levels of CD40LG in Yakutia may be associated with a higher viral load, and increased levels of IL27 in the Central region may be associated with a higher HIV viral load and incidence of diseases of the musculoskeletal system and connective tissue. Climate conditions in Yakutia, such as extremely low ambient temperatures, may influence the high prevalence of viral diseases in Yakutia—their seasonality usually peaks in winter [53,54], and antiviral nasal defense may be reduced under cold exposure [55]. The associations of musculoskeletal diseases with environmental weather conditions are quite heterogeneous [56,57]; however, high temperatures with low humidity have been associated with increased gout symptoms [58]. Interestingly, a study of search queries related to hip and knee pain showed a decrease in their number with decreasing temperatures below negative values [59]. Elevated levels of IL25 in the Central region may be associated with a higher incidence of skin diseases and circulatory diseases, and its decreased levels in Yakutia—with digestive system diseases, which are highly prevalent among the native Yakutia population [60]. Climate is one of the important factors for skin diseases—high temperatures, humidity, UV levels, and air pollution are associated with the manifestation and development of skin diseases such as psoriasis, dermatitis, and skin cancer and many others [61,62,63]. Increased levels of CCL4 in Yakutia may be associated with a lower incidence of diabetes and a higher incidence of nervous system diseases, and increased levels of CXCL10 in Yakutia, with a higher incidence of respiratory diseases. Interestingly, it is cold that may be one of the risk factors for some nervous system diseases [64], as well as exacerbate respiratory symptoms [65]. Interestingly, the immunological biomarker CXCL9, known to be the most associated with chronological age [22], is almost at the bottom of the list and varies weakly among the analyzed cohorts. This may also imply that the CXCL9-associated immunological component of aging is similar between populations. 

The observed differences in cytokine levels may be related to the genotypes of the populations studied. In particular, in a study of Canadian Aborigines living in northern Canada in a rather cold climate, higher frequencies of single-nucleotide polymorphisms of cytokines contributing to low IFNγ and TNFα production, as well as high IL-6 production, were found compared to the Caucasian population. The authors linked these results to the historical context in which these populations evolved: European populations that experienced massive epidemics were selected for genotypes that support high levels of IFNγ and TNFα expression, while the Aboriginal American population lived in areas of low population density and were less exposed to infectious diseases [66]. A similar situation could be observed for Siberian aboriginal populations. Currently, differences in the microbial environment between European populations and Siberian aboriginal populations are decreasing due to close contacts over the past century, urbanization, and globalization of lifestyle and diet. However, their genetic background may support an evolutionary immunological profile.

The immunological profile can be also influenced by chronic viral infections that do not manifest themselves but cause elevated antibody levels. In particular, chronic hepatitis rates are higher in indigenous peoples of the North American Arctic zones than in non-indigenous populations [67]. High levels of chronic hepatitis have also been found in the Yakutian population [68]. As mass vaccination against hepatitis B began less than 40 years ago, older generations have a higher incidence of chronic hepatitis without clinical manifestations. 

### 3.2. Inflammaging

Surprisingly, the SImAge inflammatory clocks did not reveal a statistically significant age acceleration between the groups. This may be due to the absence of a statistically significant difference in the most important immunomarkers for SImAge (CXCL9, CCL22, and IL6). Given the previous results indicating a consistent epigenetic age acceleration in the Yakutian group [2], it can be conjectured that the employed epigenetic clocks reflect some other mechanism of accelerated aging, activated in Yakuts and/or not strongly associated with their inflammatory status. At the same time, six out of ten cytokines in the SImAge clock (CD40LG, CXCL10, PDGFA, PDGFB, and VEGFA) are higher in Yakutia than in Central Russia, and only one, IL27, is lower in Yakutia. This remodeling of the inflammatory profile can be interpreted as an indication of a moderate trend toward stronger inflammaging in the Yakutian population. Recent studies indicate that different organs and systems may be characterized by their own clocks and different aging rates [69,70]. In this regard, our results suggest that living and long-term adaptation in extremely cold temperatures affects inflammatory and epigenetic levels differently; in particular, leading to the inflammatory clock showing no significant acceleration, while the epigenetic clock does. The underlying mechanism remains to be elucidated.

Elevated levels of pro-inflammatory immunomarkers may indicate chronic low-grade inflammation and are often examined in the context of inflammaging. These markers may be important for inflammatory clock models; in particular, the cytokine IL5 was among the most important indicators contributing positively to the iAge inflammatory age score [22] and the chemokine CXCL10 was involved in the CyClo model [27]. The release of inflammatory cytokines IL18 [18] and CCL2 [71] has also been shown to occur during inflammaging. All of the above immunomarkers have statistically higher values in residents of Yakutia, which may also indicate signs of inflammaging in this cohort.

Altogether, the uncovered pronounced regional specificity of inflammatory profiles on one hand, and only moderate traces of a stronger inflammaging among Yakuts on the other, will inspire further investigations on this topic.

### 3.3. Confounding Factors

The human immune system is dynamic and responds to both internal and external changes. Many factors can affect plasma cytokine and chemokine levels, both variable and immutable. Among them, the following groups can be distinguished:

Genetic factors: There are many studies demonstrating the influence of genetic variants on the biological mechanisms regulating cytokine levels, as well as on individual differences in response to pathogens [72,73,74,75,76]. 

Evolutionary mechanisms: Studies of evolutionary mechanisms have shown their influence on cytokine production and inheritance of cytokine pathways [77,78], and the evolutionarily optimal immune response arises from the epidemiologic environment, life cycle, and demography of the whole organism [79].

Climate factors: The immune system in general and mechanisms related to cytokine production in particular can be influenced by a variety of climatic factors, including ambient temperature [80], UV exposure [81], humidity [82], climate change [83,84], water and air pollution (sanitation problems in Yakutia were previously reported [85]).

Unhealthy habits: Cytokine profiles can be altered by constant negative exposures of the body, including from unhealthy habits such as smoking and alcohol consumption [86,87,88,89,90].

Lifestyle: The conditions of a person’s daily life also imprint on immune status, such as physical activity, nutrition, and sleep quality. Diet, gut microbiome, and the immune system are closely linked, and studying the influence of an individual’s nutritional type on their inflammatory status is of particular interest [78,86,91]. The cytokine profile and immune response can be influenced by regular exercise, and even its effectiveness can be monitored by the levels of individual cytokines [92,93]. Sleep regime can also be included in this category, as sleep deprivation, in particular, can affect levels of inflammatory immunomarkers [94].

Also potentially influencing immune status are access to medical care, socioeconomic status, cultural background, and other factors. Thus, there is a wide range of factors that can influence plasma cytokine and chemokine levels. A more in-depth study with additional cohort information is of particular interest so that the influence of different cofactors can be clearly separated.

### 3.4. Further Research Directions

Further studies could take several directions. First, it is of clear interest to test the other models of inflammatory clocks, as well as to conduct a detailed investigation of the divergence of immunological and epigenetic clocks, targeting epigenetic markers associated with inflammatory status. Second, it is appealing to expand data collection in Yakutia, also recording additional information on potential confounders, including a comparative analysis of inflammatory status in extreme and non-extreme climates. Third, it is challenging to broaden the geographic and evolutionary genetic context to include other northern areas, such as northern Canada and Scandinavia, Alaska, Greenland, and high mountainous areas, like the Andes and Tibet. Another promising issue is to address the short-term adaptation from the proposed perspective.

### 3.5. Limitations

Several limitations have to be addressed. 

Firstly, many specific details regarding phenotype and daily living conditions are not available, due to the inherent difficulties associated with data collection in a remote area. Factors potentially affecting immunologic profiles in extremely cold climates require more detailed investigation.

There is an imbalance in the available data, given that fewer Yakut individuals participated. However, this was taken into account when building the deep neural network classifier.

The sample size of the data analyzed in this paper is limited, and the results should be cautiously extrapolated to entire populations: (1) diversity and variability may be much higher in the whole population than in a small sample; (2) an outlier in a small sample may be an inlier in a large sample; (3) without information on factors that may affect cytokine levels, there may be bias in the data; (4) there may not be enough data to reach statistical significance for individual cytokines. The results of the classifier may change if the sample size increases (either due to samples for these regions or data for other regions).

Immunological differences are the result of multiple factors, and climate is but one among them; the detailed influence of others requires further study with more data. 

Only two regions from the same country were compared. It would be of interest to extend the analysis to include data from other parts of the world when similar data are available in open access. It would then be possible to analyze the effects of different types of climatic extremes on the immunological profiles and to identify the similarities and differences between them.

## 4. Materials and Methods

### 4.1. Data Collection

Participants were recruited as volunteers who responded to an invitation to participate in the study following screening against the exclusion criteria. Efforts were made to reduce heterogeneity in age and sex composition. All participants signed an informed consent form explaining the purpose of the project, voluntary participation, confidentiality, potential inconvenience, and details of the study procedure. Participation in the study was not financially rewarded. All procedures performed in this study were free of charge to volunteers. All participants filled out a consent form for the processing of personal data, taking into account the principle of confidentiality, assuming accessibility only to the research team and presentation of data in a common array. This study was approved by the local ethical committee of Nizhny Novgorod State University. All study procedures were conducted in accordance with the 1964 Declaration of Helsinki and its subsequent amendments. Exclusion criteria included chronic diseases in the acute phase, oncologic diseases, acute respiratory viral infections, and pregnancy. Diseases affect the levels of pro-inflammatory and anti-inflammatory markers in the immunologic profile and may be heterogeneous in their manifestations, which could have influenced the results.

The assay was performed on plasma using K3-EDTA anticoagulant, without hemolysis and lipemia. Plasma was thawed, centrifuged (3000 rpm, 10 min) to remove debris, and 25 μL was collected in duplicate. Plasma with antibody-immobilized beads was incubated with agitation on a shaker overnight (16–18 h) at 2–8 °C. The Luminex^®^ assay was run according to the manufacturer’s instructions using a 46-plex human cytokine panel (EMD Millipore Corporation, Darmstadt, Germany, HCYTA-60 K-PX48). The assay plates were measured using Magpix (Milliplex MAP). Data acquisition and analysis were performed using the standard MAGPIX^®^ software program set xPONENT version 4.2. Data quality was examined based on the following criteria: the standard curve for each analyte had a 5P R2 value > 0.95. To pass the assay technical quality control, the results for the two controls in the kit needed to be within the 95% confidence interval (CI) provided by the vendor for > 40 analyses tested. No further tests were performed on samples with results out of range low (<OOR). Samples with results out of range high (>OOR) or greater than the standard curve maximum value (SC max) were not tested at higher dilutions.

Data quality control was performed for both groups (Central Russia and Yakutia). Only immunologic parameters, which had no missing values in the majority of samples, were kept for analysis, which left us with 32 features. Data were also examined for the total number of outliers. Outliers were defined by IQR (values of cytokine levels outside the interval [Q1 − 1.5 × IQR; Q3 + 1.5 × IQR] were defined as outliers). Next, we counted the number of features for which a sample was an outlier with a threshold of 25% (if at least a quarter of the features in a particular sample were outliers, the sample was considered an outlier). There were no such samples, so all were included in the analysis.

### 4.2. Statistical Analysis

The difference in the distribution of immunological biomarker levels in the studied groups was tested using the Mann–Whitney U-test [95]. This is a nonparametric test for comparing the results of two independent groups, which is used to test the probability that two samples come from the same population, with a two-sided null hypothesis that the two groups are not the same. The variance of the distributions of biomarker levels was tested using the Levene test [96]. This is a nonparametric test, with a null hypothesis that the variances(relative to median) are equal in the two groups. All obtained *p*-values were FDR-corrected according to the Benjamini–Hochberg procedure [97].

### 4.3. Deep Neural Network Classifier

Immunological data is an example of a tabular data format, with columns containing immunological biomarkers, rows containing participants, and a numerical value in each cell representing the level of a particular immunological biomarker in a particular sample. The target variable here is the cohort (Yakutia or Central Russia), and we faced a binary classification task. Already extracted features and the absence of spatial relationships between them (like in images and texts, for example) characterize tabular data, so specialized neural network architectures were developed to handle them. 

To build a deep classifier that can separate representatives of Yakutia and Central Russia by their immunological profile, we used lightweight architectures such as multilayer perceptron (MLP), TabNet [98], Feature Tokenizer and Transformer (FT-Transformer) [99], Gated Adaptive Network for Deep Automated Learning of Features (GANDALF) [100] and Deep Abstract Network (DANet) [101]. MLP is one of the simplest neural network architectures composed of several dense layers. TabNet consists of sequential modules, each of which implements a sequential attention mechanism that selects the most relevant features. FT-Transformer is an adaptation of the transformer architecture to tabular data. GANDALF uses a new tabular processing unit with a gating mechanism and built-in Gated Feature Learning Unit (GFLU) feature selection. DANet is focused on abstract layers, the main idea of which is to group the correlated features and create higher-level abstract features from them. 

Before training the classifier, all data were divided into 2 parts: 80% for training/validation and 20% for independent testing. When training the model, cross validation is used, i.e., sequential division of the data into training and validation samples at a ratio of 3 to 1, until all data are in the role of training and validation sets. Cross-validation is necessary to determine the best combination of model hyperparameters to ensure the highest classification accuracy. Since in the analyzed data there are more participants from the Central region than participants from Yakutia, class imbalance techniques are required to build a correct classifier. Weighted sampler ensures that the original class distribution is preserved in the training, validation and test sets. All quality metrics used for the machine learning model have macro averaging, which consists of simple averaging without weighting factors, which allows classes to be considered equal despite an imbalance in numbers. To avoid the model overfitting, we controlled the losses during the training and validation data to stop the training early enough to prevent the model from memorizing the data.

The final model results were calculated on a test set (not involved in any way in the model training). A visual representation of the classifier results is the confusion matrix, which shows the number of correctly and incorrectly classified samples of all types. For a comprehensive assessment of the model quality, we considered the following metrics: accuracy, F-1 score, precision, recall, specificity, AUROC, and Cohen’s kappa. Their formulas are given below. All of these metrics were macro-averaged:

$$Accuracy=\frac{TP+TN}{TP+TN+FP+FN},\;F_{1}=2\frac{Precision\times Recall}{Precision+Recall},\;Precision=\frac{TP}{TP+FP},\;Recall=\frac{TP}{TP+FN},\;Specificity=\frac{TN}{TN+FP},\;Cohen’skappascore=\frac{2\times \left(TP\times TN-FN\times FP\right)}{\left(TP+FP\right)\times \left(FP+TN\right)+\left(TP+FN\right)\times \left(FN+TN\right)},$$

where TP is true positive, FP is false positive, TN is true negative, and FN is false negative.

Deep neural network architectures are usually black boxes with opaque decision-making principles. However, there is active development of explainable artificial intelligence approaches that can explain why models make certain decisions, in particular, when classifying samples. One of the most common approaches is SHAP values, which use game theory principles [102]. They are calculated for each sample and each feature and show how a particular value changes the model’s final prediction. As a result, SHAP helps to identify the features that contribute most to the final classifiers and which feature values contribute to the probability of predicting a particular class.

## 5. Conclusions

The study presents the first report of cytokine profile data from the Yakut population and a comparative analysis with the corresponding data from the Central Russian population. Two main questions are addressed: differences in cytokine levels (by statistical tests and by a deep neural network classifier) and the significance of inflammatory age acceleration in Yakuts. Cytokines with statistically significant mean and variance differences were found. Individual inflammatory profiles between the two groups were quite accurately discriminated by an explainable deep neural network classifier. The difference in biological age acceleration between the two cohorts on the SImAge clock was not statistically significant, although higher levels of inflammatory cytokines positively correlating with age in Yakuts may indicate a tendency toward inflammaging. Generally, the influence of environmental factors on aging and age-related diseases is under active investigation within the concept of the exposome. Inflammaging is one of the main mechanisms of aging, and studying the contribution of environmental factors on inflammaging appears challenging. The results of the study are one of the first steps in understanding this relationship, and further research may focus on other conditions besides extreme cold, such as a hot, humid and dry climate, as well as low pressure and oxygen levels at high altitudes.

## Figures and Tables

**Table 1:** Summary of the most different cytokines and respective key associations.

Cytokine	Key Associations	Relation to the Studied Regions	References
CD40LG	Inflammation associated with viral infection	Higher viral load in Yakutia	[34]
	Increases in patients with Viliui encephalomyelitis and in healthy family members	Viliui encephalomyelitis occurs only in the indigenous population of Yakutia	[38]
IL27	Increases in patients with rheumatoid arthritis, systemic lupus erythematosus, multiple sclerosis	Less common in Yakutia	[42]
	Negatively correlated with HIV viral load	Higher HIV incidence in Yakutia	[42]
IL25	Activated eosinophils and basophils in allergy	Fewer cases of allergy in Yakutia	[43]
	Development and severity of skin diseases (dermatitis, psoriasis)	Less common in Yakutia	[43]
	High level is a biomarker of cardiovascular disease risk	Less common in Yakutia	[43,44]
CCL4	Decreases in patients with type 1 and type 2 diabetes	Fewer cases of both types in Yakutia	[47]
CXCL10	Respiratory symptoms in viral diseases	Higher viral load and more respiratory diseases in Yakutia	[50]
	COVID-19 and its complications	Higher levels of COVID-19 in Yakutia	[52]

## Data Availability

The original contributions presented in this study are included in the Supplementary Material. Further inquiries can be directed to the corresponding author.

## Supplementary Materials

The following supporting information can be downloaded at: https://www.mdpi.com/article/10.3390/ijms252413741/s1.
